# Knockdown of *Kmt2d* leads to growth impairment by activating the Akt/β-catenin signaling pathway

**DOI:** 10.1093/g3journal/jkad298

**Published:** 2024-01-23

**Authors:** Huakun Shangguan, Xiaozhen Huang, Jinduan Lin, Ruimin Chen

**Affiliations:** Department of Endocrinology, Genetics and Metabolism, Fuzhou Children’s Hospital of Fujian Medical University, Fuzhou 350000, China; Department of Endocrinology, Genetics and Metabolism, Fuzhou Children’s Hospital of Fujian Medical University, Fuzhou 350000, China; Department of Endocrinology, Genetics and Metabolism, Fuzhou Children’s Hospital of Fujian Medical University, Fuzhou 350000, China; Department of Endocrinology, Genetics and Metabolism, Fuzhou Children’s Hospital of Fujian Medical University, Fuzhou 350000, China

**Keywords:** KMT2D, Akt/β-catenin, growth, chondrocyte

## Abstract

The *KMT2D* variant–caused Kabuki syndrome (KS) is characterized by short stature as a prominent clinical characteristic. The initiation and progression of body growth are fundamentally influenced by chondrocyte proliferation. Uncertainty persists regarding the possibility that KMT2D deficiency affects growth by impairing chondrocyte proliferation. In this study, we used the CRISPR/Cas13d technique to knockdown *kmt2d* in zebrafish embryos and lentivirus to create a stable *Kmt2d* gene knockdown cell line in chondrocytes (ATDC5 cells). We also used CCK8 and flow cytometric studies, respectively, to determine proliferation and cell cycle state. The relative concentrations of phosphorylated Akt (ser473), phosphorylated β-catenin (ser552), and cyclin D1 proteins in chondrocytes and zebrafish embryos were determined by using western blots. In addition, Akt inhibition was used to rescue the phenotypes caused by *kmt2d* deficiency in chondrocytes, as well as a zebrafish model that was generated. The results showed that a knockdown of *kmt2d* significantly decreased body length and resulted in aberrant cartilage development in zebrafish embryos. Furthermore, the knockdown of *Kmt2d* in ATDC5 cells markedly increased proliferation and accelerated the G1/S transition. In addition, the knockdown of *Kmt2d* resulted in the activation of the Akt/β-catenin signaling pathway in ATDC5 cells. Finally, Akt inhibition could partly rescue body length and chondrocyte development in the zebrafish model. Our study demonstrated that KMT2D modulates bone growth conceivably via regulation of the Akt/β-catenin pathway.

## Graphical Abstract

**Graphical abstract:**
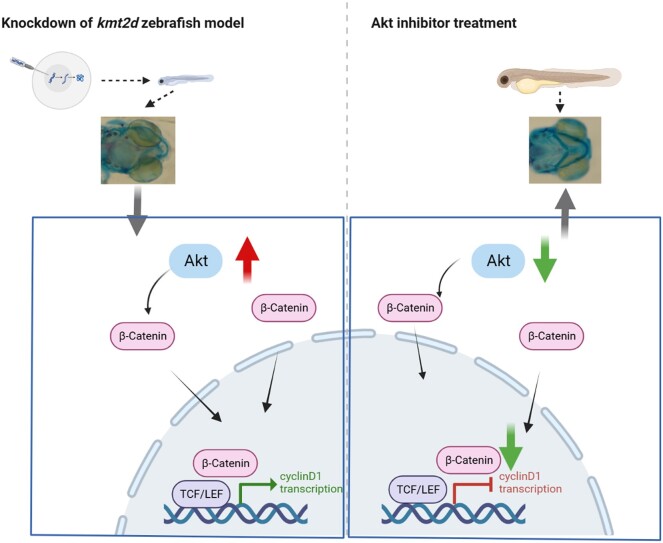

## Introduction

Yoshikazu Kuroki and Norio Niikawa initially named and documented Kabuki syndrome (KS; OMIM#147920) in the early 1980s (Adam *et al*. 2019). About 70% of people with KS have genetic variations in the *KMT2D* and *KDM6A* genes, with the former being more prevalent. As a histone (H3) lysine methyltransferase protein, the KMT2D encodes an epigenetic factor that controls chromatin shape and gene transcription by methylating lysine 4 on histone H3 (Jefri *et al*. 2022). A distinctive facial characteristic of patients with KS was the eversion of the lateral third of the lower eyelids, as well as long palpebral fissures, a flattened nasal tip, and enormous ears (Usluer *et al*. 2022). Short stature, cerebral impairments, cardiac issues, small hands, and clinodactyly of the fifth finger are additional primary clinical traits of KS (Adam *et al*. 2019).

The cause of the short stature in KS is still unresolved. Reduced body length in all 3 strains of *Kmt2d*^−/+^ mice that were separately developed (Fahrner *et al*. 2019; Huisman *et al*. 2021). The *Kmt2d*^−/+^ mouse exhibits a growth delay phenotype in one of the strains and has impaired growth hormone–releasing hormone neuron function (Huisman *et al*. 2021). Nonetheless, many documented accounts of growth hormone deficiency in *KMT2D* patients are scant (van Montfort *et al.* 2021). Chondrocyte proliferation contributes to the initiation and development of body growth. The enhanced chondrocyte differentiation caused by the loss of *Kmt2d* has also been linked to growth impairment (Fahrner *et al*. 2019). The question whether *KMT2D* deficiency inhibits linear growth by impairing chondrocyte proliferation is an unsettled one.

Prokaryotes’ adaptive immune system, which was employed to protect against phage viral invasion, was encoded by CRISPR/Cas loci. Prokaryotes can precisely recognize gene sequences that resemble phages or other invaders using the CRISPR system, and then they can employ CRISPR-associated proteins (Cas) to target these sequences for destruction (Knott and Doudna 2018). A novel technique called CRISPR/Cas13 uses RNA editing to knockdown genes without changing the genome (Cox *et al*. 2017). Six distinct Cas13 variants have been found so far, and Cas13d, one of the smallest single-effector Cas enzymes, has been isolated and identified (Zhang *et al*. 2018). Zebrafish (*Danio rerio*) serve as an adaptive and reliable model for genetic and developmental research. The CRISPR/Cas13d technique was initially used by Kushawah to examine gene activity in a zebrafish model in 2020, and the results demonstrated remarkable efficiency and specificity (Kushawah *et al*. 2020).

In this study, we investigated the molecular mechanisms behind the *KMT2D* gene deficiency–related short stature. Our results found that KMT2D modulates growth possibly via activating the Akt/β-catenin signaling pathway.

## Materials and methods

### Zebrafish maintenance and cell cultivation

The wild-type AB strain of zebrafish used in this experiment was obtained from the laboratory of School of Biological Engineering, Fuzhou University. Adult wild-type AB strains of zebrafish were maintained under standard laboratory conditions (28.5°C and 14 h/10 h light–dark cycle). Live images were captured using an SMZ 1500 stereomicroscope (Nikon), and body length was determined by image-based morphometric analysis using NIS-Elements D3.1 software.

The murine chondrogenic cell line ATDC5 was obtained from an American-type culture collection. The cell line was incubated in a Dulbecco’s modified eagle medium (DMEM) complete medium (Thermo Scientific, Waltham, MA, USA) supplemented with 10% fetal bovine serum (Gibco, Thermo Scientific) and 1% penicillin/streptomycin solution (Solarbio Science & Technology, Beijing, China) at 37°C in an atmosphere containing 5% CO_2_ in air.

### Evolutionary comparison of KMT2D proteins

Sequences of proteins belonging to the KMT2D families were identified in genomes by using the NCBI (https://www.ncbi.nlm.nih.gov/) and Ensembl (http://www.ensembl.org/index.html). For the analysis of KMT2D, all data were collated and mapped onto a phylogenetic tree by MEGA 6.0.

### Guide RNA and Cas13d mRNA generation

Briefly, 3 guide RNAs (gRNAs) were designed to disrupt the *kmt2d* gene in zebrafish with the following sequences: gRNA1: CTCAGCAGAGCTTGGCTGGGAGG; gRNA2: CGGCCTATCAAAACTGAACCTGG; gRNA3: CTGCTAAGCTCCTCTTCGCTGGG.

A DNA template to generate gRNA mRNA was generated by fill-in PCR. For RNA synthesis, PCR amplicons were purified and used as templates using T7 RNA polymerase (MAXIscript T7 Kit, Invitrogen) according to the manufacturer’s instructions. Cas13d were PCR-amplified using primers (Cas13d-SP6-kozak-F: 5′- ATTTAGGTGACACTATAGAAGTGCCGCCACCATGAGCCCCAAGAAGAAG-3′ and Cas13d-WPRE-R: 5′-ATTGCTACTTGTGATTGCTCCATG-3′) and also using addgene plasmid # 109049 (a gift from Pr. Lisa Li) as the template. Then, the Cas13d mRNA was synthesized using the mMACHINE SP6 Transcription Kit (Invitrogen). To increase mRNA depletion, the 3 gRNAs targeting the *kmt2d* gene were co-injected. One nanoliter containing 200 ng of Cas13d mRNA and 100 ng of gRNA mRNA were co-injected into the 1-cell stage zebrafish embryos.

### Zebrafish Alcian blue and Alizarin red staining zebrafish staining

The cartilage and bone of zebrafish at 5-day postfertilization (dpf) were double-stained with Alizarin red/Alcian blue at the same time, according to a previously reported method to observe the bone morphology (Nagy *et al*. 2009). Images were analyzed under a dissecting microscope (Zeiss, German).

### Generation of *Kmt2d* knockdown cells by short hairpin RNA

The sequences of the short hairpin RNA (shRNA) were cloned into the lentiviral vector pLKO-puro, which could express shRNA and inhibit the expression of a specific gene by the RNA interference mechanism. Lentiviral particles were produced in HEK293T cells, and subsequently, the ATDC5 cells were transduced. The sequences of the shRNA targeting the *Kmt2d* gene were as follows:

- *Kmt2d*-sh1: 5′-CCTTGCCAGTTCACCATTAAT-3′
- *Kmt2d*-sh2: 5′-GTTCATCGAGTTGCGACATAA-3′
- *Kmt2d*-NC: 5′-TTCTCCGAACGTGTCACGTAA-3′

### qRT-PCR and western blot

In the study, 15 embryos per biological replicate were obtained at 6 h postfertilization (hpf), and the expression level of *kmt2d* mRNA was validated by qRT-PCR. We used the FreeZol Reagent kit (Vazyme, China) to extract the total RNA of zebrafish embryos or cells and reverse-transcribed it into cDNA following the manufacturer’s protocol. The expression level of mRNA was assessed by qRT-PCR using specific primers and ChamQ SYBR qPCR Master Mix (Vazyme). The level of mRNA was calculated by using the $2^{-\Delta \Delta C_{t}}$ method. Because the level of *cdk2ap2* mRNA does not change during the first 6 h of zebrafish development, we used it as a control when measuring the mRNA level in zebrafish (Kushawah *et al*. 2020). The quantitative PCR primers are as follows:

- *kmt2d*-F(zebrafish): 5′-GCACGAGTATGTCCAAAGCC-3′
- *kmt2d*-F(zebrafish): 5′-CAGTCGACGGTACTGAGAGG-3′
- *cdk2ap2*-F(zebrafish): 5′-AGGATCTTGTGGCTCTTCTCCATCAC-3′
- *cdk2ap2*-R(zebrafish): 5′-TTTCACGGCTCATCTCCTCAATGAC-3′
- *Kmt2d*-F(mus): 5′-ACCGAGTGGAAGAACAATGTG-3′
- *Kmt2d*-R(mus): 5′-CGAATGATGGTGCCGATGTA-3′
- *Gapdh*-F(mus): 5′-CTCCTGCACCACCAACTG CTTAG-3′
- *Gapdh*-R(mus): 5′-GACGCCTGCTTCACCACC TTC-3′

The primary antibodies of rabbit monoclonal antihistone H3 (monomethyl K4; Abcam, ab8895), antihistone H3 (trimethyl K4; Abcam, ab8580), histone H3K4me3 (trimethyl Lys4) antibody (GeneTex, GTX128954), rabbit monoclonal anti-p-Akt (ser473; Cell Signaling, #4060), Akt (phospho Ser473) antibody (GeneTex, GTX11901), rabbit monoclonal anti-p-β-catenin (ser552; Cell Signaling, #5651), and rabbit monoclonal anti-cyclin D1 (Abcam, ab16663) were used to perform western blot analysis under standard protocol. Protein expression levels were quantitated by using Photoshop CS4 software (Adobe Company, USA).

### CCK-8 assay and cell cycle

The cell counting kit-8 (CCK-8) assays were conducted using the CCK-8 kit (Beyotime, China) to measure the proliferation of ATDC5 cells. Flow cytometry was used to detect changes in the cell cycle through the Cell Cycle Analysis Kit (Beyotime). These experiments were performed following the manufacturer’s protocol and in triplicate.

### Statistical analysis

The experimental data were statistically analyzed using GraphPad Prism 8 software. Data were expressed as mean ± standard deviation (SD), and the *t*-test was used for intergroup comparison. A score of *P* < 0.05 was considered statistically significant.

## Results

### Zebrafish *kmt2d* is highly conserved across multiple species

An analysis of multiple sequence alignments can help generate essential clues for genomic data analysis and reveal functionally important regions in proteins. KMT2D’s enzymatic activity is controlled by its SET domain—zebrafish, humans, and mice differ in only 7 amino acids (Supplementary Fig. 1a). Additionally, we used MEGA 6.06 to perform an evolutionary tree analysis, and the findings revealed that zebrafish *kmt2d* had the most prominent evolutionary connections to Bufo gargarizans, humans, and mice (Supplementary Fig. 1b). The findings suggest that zebrafish *kmt2d* is highly conserved, particularly in the catalytic domain and across a variety of species.

### The influence of *kmt2d* deficiency in zebrafish embryos

**Fig. 1.**
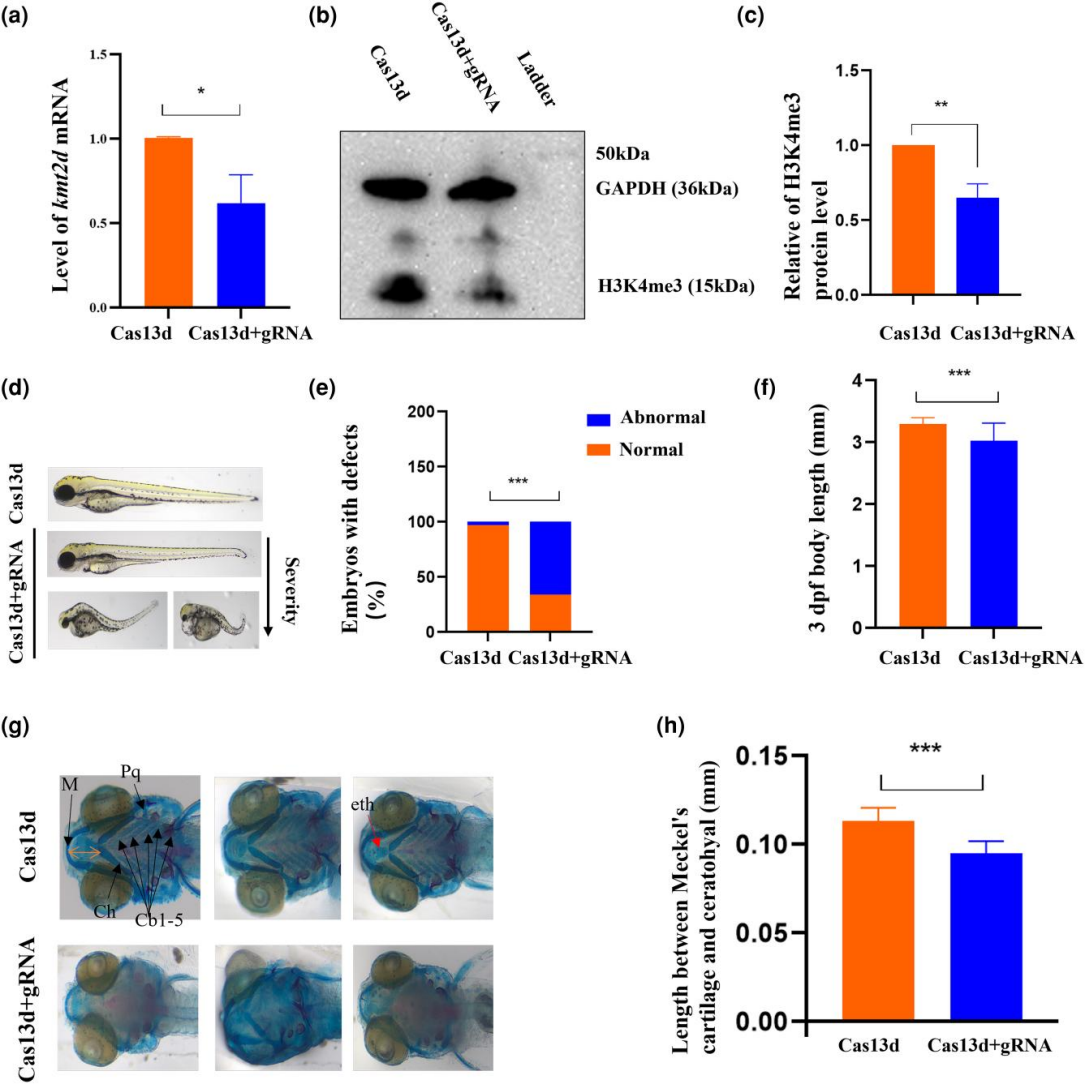
The influence of *kmt2d* deficiency in zebrafish embryos. a) Fifteen embryos per biological replicate were collected for zebrafish qRT-PCR at 6 h post injection. b and c) The level of the H3K4me3 protein. The band density was quantified and expressed as the relative gray value (compared with the control) by arbitrarily setting the control value as 1. Data were pooled from 3 independent experiments, and the results were represented as mean ± SD. d and e) Abnormal phenotypes including small eyes, curved body, pericardial effusion, mandibular deformity, slight tail curvature, and shortened yolk extension were observed in 3 dpf stage. *n* = 35 for each group. f) Body length was reduced in the *kmt2d* knockdown zebrafish. *n* = 19 for each group. g) Dysplasia formation in Meckel’s cartilage, ceratohyals, and ethmoid bones in the embryos with a co-injection of Cas13d mRNA and 3 gRNAs. *n* = 7 for each group. h) Quantification of length between Meckel’s cartilage and ceratohyal. *kmt2d* knockdowns were shorter when compared with those of control embryos. *n* = 7 for each group. eth, ethmoid plate; Cb, ceratobranchial; Ch, ceratohyal; M, Meckel’s cartilage; Pq, palatoquadrate. Scale bar: b) 1,000 μm; e) 500 μm. *P < 0.5, **P < 0.001, ***P < 0.0001.

Zebrafish larvae were utilized to assess the effects of *kmt2d* deficiency in order to investigate the role of KMT2D on growth. The CRISPR/Cas13d system was used to knock down the *kmt2d* gene in zebrafish (Supplementary Fig. 2; Hernandez-Huertas *et al*. 2021). By at least 6 hpf, the CRISPR/Cas13d system triggered *kmt2d* mRNA degradation (Cas13d alone vs Cas13d and gRNAs, *P* < 0.05, Fig. 1a). Concurrently, the H3K4me3 level was decreased in these embryos with a *kmt2d* deficit (Fig. 1b and c). The rate of morphological abnormalities was higher when Cas13d and 3 gRNAs targeting the *kmt2d* mRNA were co-injected into 1 cell-stage zebrafish embryo (65.7 vs 2.9%, *P* < 0.0001; Fig. 1e). Small eyes, a curved body, pericardial effusion, a mandibular deformity, a modest tail curvature, and a reduced yolk extension were among the morphological imperfections (Fig. 1d). In terms of growth, zebrafish in the knockdown group had a shorter body length than that in the control group (3.023 ± 0.2856 vs 3.298 ± 0.1003, *P* < 0.0001; Fig. 1f).

Defects in bone development are pathological causes of short stature (Lui 2020). Because of its highly conserved endochondral ossification, the zebrafish are ideally suited for the genetic causes of short stature (Garcia *et al*. 2016). To identify skeletal alterations, the entire skeleton is stained with Alizarin red and Alcian blue. In comparison with embryos injected with Cas13d mRNA alone at 5 dpf, those with a co-injection of Cas13d mRNA and 3 gRNAs targeting *kmt2d* (6/7; 85.7%) failed to form the cranial bone, such as the ethmoid plate (Fig. 1g, red arrow). Additionally, visceral cartilages in zebrafish with *kmt2d* deficiency were considerably impacted (Fig. 1g, black arrow). The structures of the ceratobranchial that support the delicate gill tissues (Fig. 1g; Cb1-5) were completely absent in *kmt2d* knockdowns (7/7; 100%). The cartilage, which forms the lower jaw, was deformed (Fig. 1f, Meckel’s cartilage; 42.8%) or lacking (28.6%; Fig. 1g, palatoquadrate and ceratohyal). Meckel’s cartilage and the ceratohyal had an average length of 0.11 ± 0.007 mm in normal embryos (Fig. 1h, orange arrow; *n* = 7), whereas *kmt2d* knockdowns had a much shorter configuration with an average length of 0.09 ± 0.006 mm (Fig. 1h; *n* = 7). The incorrect use of the staining procedure led to eye defects in the *kmt2d* deficient embryos depicted in Fig. 1g. Co-injective of full-length *kmt2d* cDNA with Cas13d plus 3 gRNAs would be instructive to ascertain whether the phenotypes are caused by a downregulation of *kmt2d*. However, it proved difficult since the zebrafish kmt2d gene comprises 53 exons and a 17.6 kb mRNA that codes for a 362.7 kDa protein, making the extraction of full-length cDNA difficult. Overall, these findings showed that *kmt2d* was necessary for endochondral ossification to occur normally in zebrafish.

### *Kmt2d* knockdown promoted ATDC5 cell cycle progression and proliferation in vitro

**Fig. 2.**
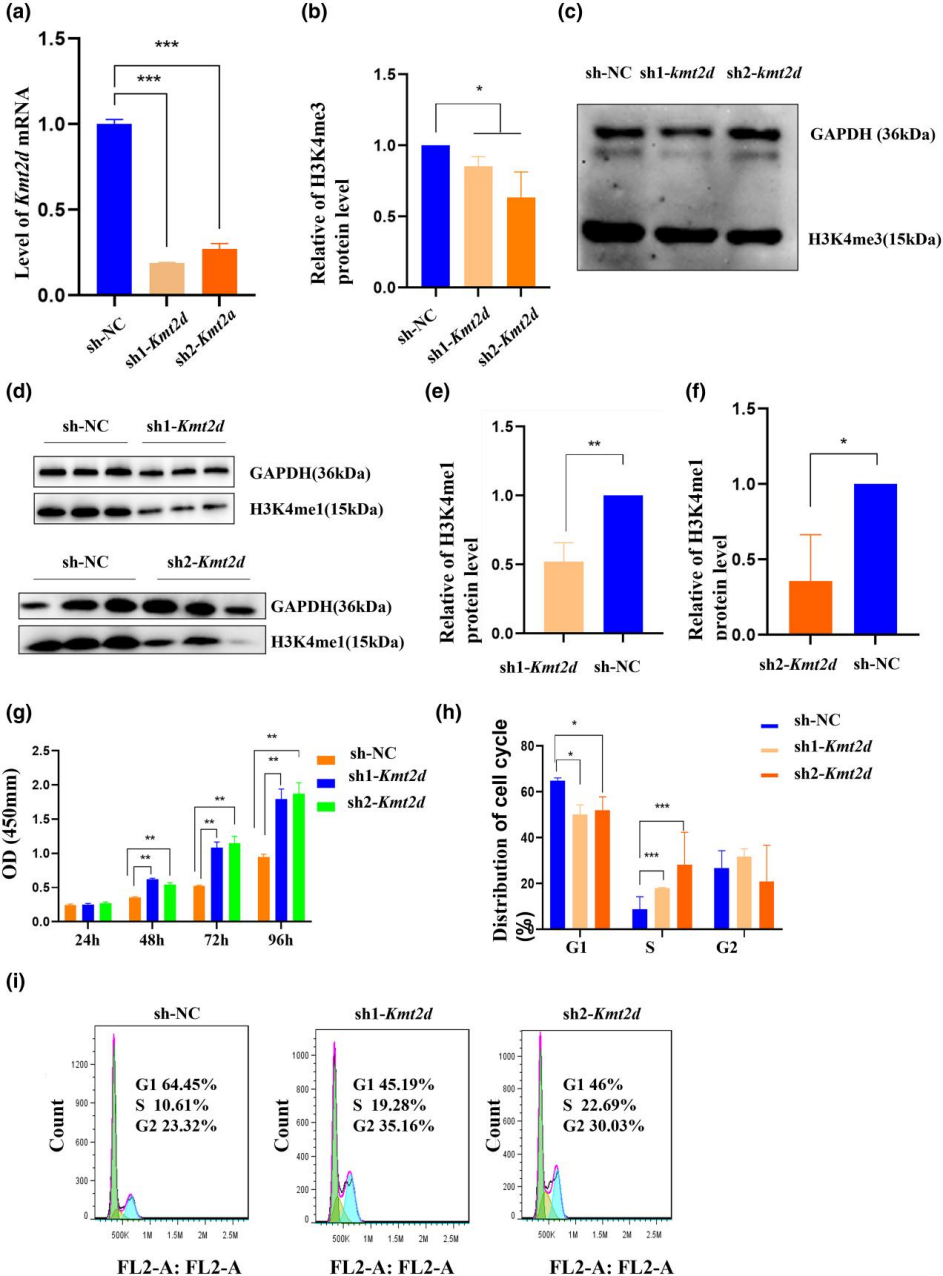
*Kmt2d* knockdown promoted ATDC5 cell cycle progression and proliferation in vitro. a) The level of *Kmt2d* mRNA in the ATDC5 cells. b–f) The levels of H3K4me1 and H3K4me3 proteins. The band density was quantified and expressed as the relative gray value (compared with the control) by arbitrarily setting the control value as 1. Data were pooled from 3 independent experiments, and the results were represented as mean ± SD. g) CCK-8 assay was performed to measure the proliferation of ATDC5 cells. The proliferation of the cells was enhanced in the knockdown of *Kmt2d* groups. h and i) Cell distribution in the G1, S, and G2/M phases of the sh-NC cells compared with those of the sh-*Kmt2d* cells. *P < 0.05, **P < 0.001, ***P < 0.0001.

**Fig. 3.**
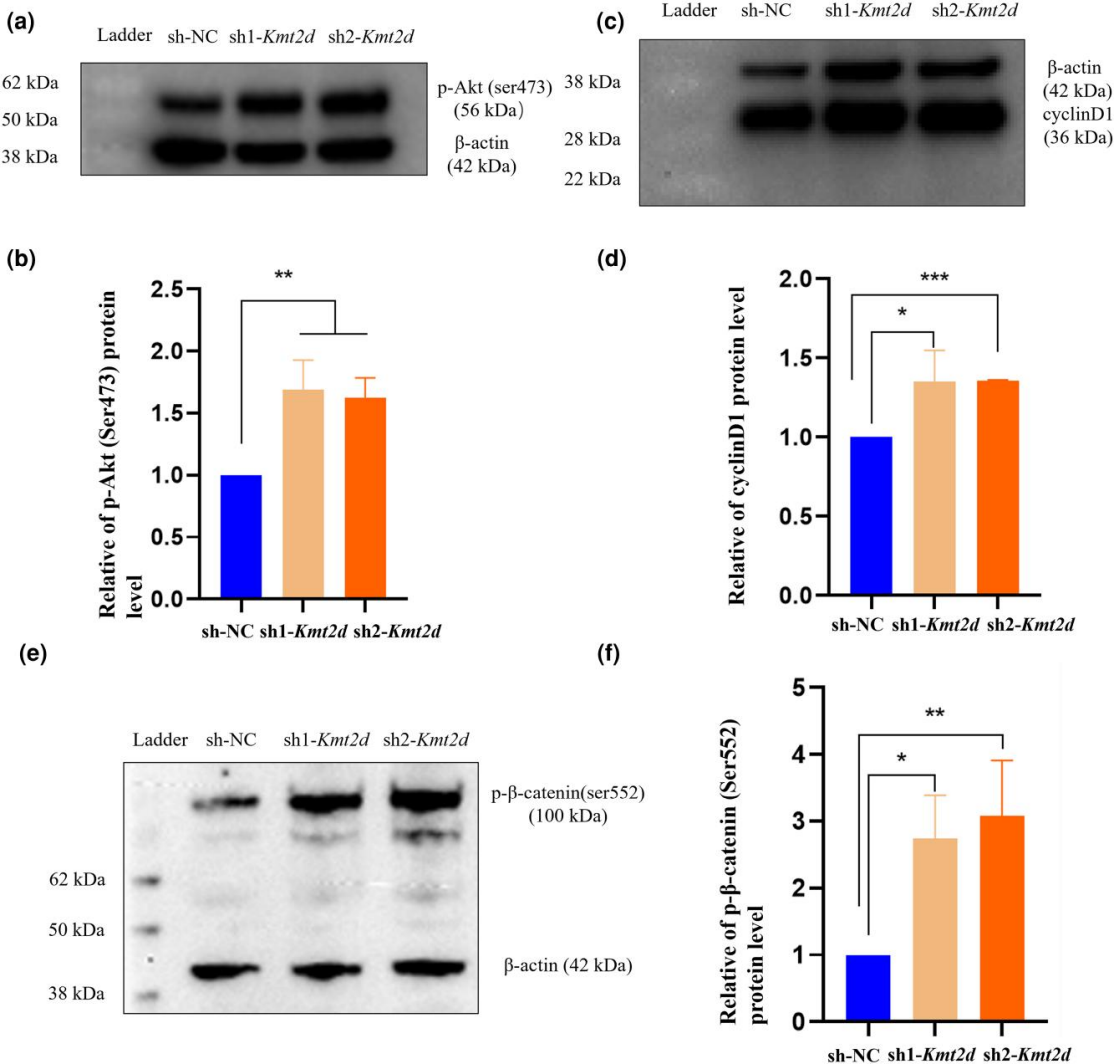
*Kmt2d* knockdown resulted in Akt/β-catenin pathway activation in vitro. a and b) The relative p-Akt (Ser473) protein level decreased in the knockdown group. c and d) The relative p-β-catenin (Ser552) protein level decreased in the knockdown group. e and f) The relative cyclin D1 protein level decreased in the knockdown group. The band density was quantified and expressed as the relative gray value (compared with the control) by arbitrarily setting the control value as 1. Data were pooled from 3 independent experiments, and the results were represented as mean ± SD. *P < 0.05, **P < 0.001, ***P < 0.0001.

Increased proliferation may have an impact on the formation of bones and cartilage (Lui 2020). The ATDC5 cell line, isolated from mouse teratocarcinoma cells, is a chondrogenic cell line with a sequential process analogous to chondrocyte differentiation. Therefore, it is considered as a promising *in vitro* model to explore signaling pathways related to bone growth (Yao and Wang 2013). The proliferation of ATDC5 cells was then examined in relation to KMT2D. We created an ATDC5 cell line with *Kmt2d* permanently silenced by transfecting it with lentiviruses that expressed 2 *Kmt2d*-specific shRNAs, as shown in Fig. 2a. Additionally, the levels of both the H3K4me1 and the H3K4me3 proteins were steadily declining (Fig. 2b–f). The *kmt2d* knockdown group had increased proliferation of undifferentiated ATDC5 cells (Fig. 2g), which may be attributed to the increased expression of cell cycle–related molecular markers (cyclin D1, Fig. 3e). Previous research suggested that faulty cell cycle progression led to unchecked cellular proliferation and begot abnormal chondrogenesis (Beier 2005). We observed that a reduced expression of *Kmt2d* in ATDC5 cells accelerated the G1/S transition and increased cell cycle progression (Fig. 2h and i). Together, our findings suggest that KMT2D may play a crucial role in cell proliferation and cycle progression throughout the development of cartilage.

### Knockdown of *Kmt2d* resulted in the Akt/β-catenin pathway activated in vitro

We have demonstrated a connection between *KMT2D* and the control of cell proliferation and cycle progression. Additionally, we propose that Akt signaling controls chondrocyte proliferation, and altering the activation of Akt may cause growth delay in a mouse model (Fukai *et al*. 2010). Also, of relevance, Akt activity can be fully activated by phosphorylation at serine 473 (Persad *et al*. 2001). In this study, western blot was used to detect p-Akt (ser473), and the results suggested that *Kmt2d* knockdown increased the expression level of p-Akt (ser473) compared with that of the control group (Fig. 3a and b). A β-catenin-dependent upregulation of cyclin D1 transcription and faster cell cycle entry can occur when Akt directly phosphorylates β-catenin at serine 552 (Han *et al*. 2021). Correspondingly, the knockdown group exhibited increased expression levels of p-β-catenin (ser552) and its downstream target gene cyclin D1, when compared with the control group (Fig. 3c–f). The findings herein suggest that the knockdown of the *Kmt2d* gene leads to the activation of the Akt/β-catenin signaling pathway.

**Fig. 4.**
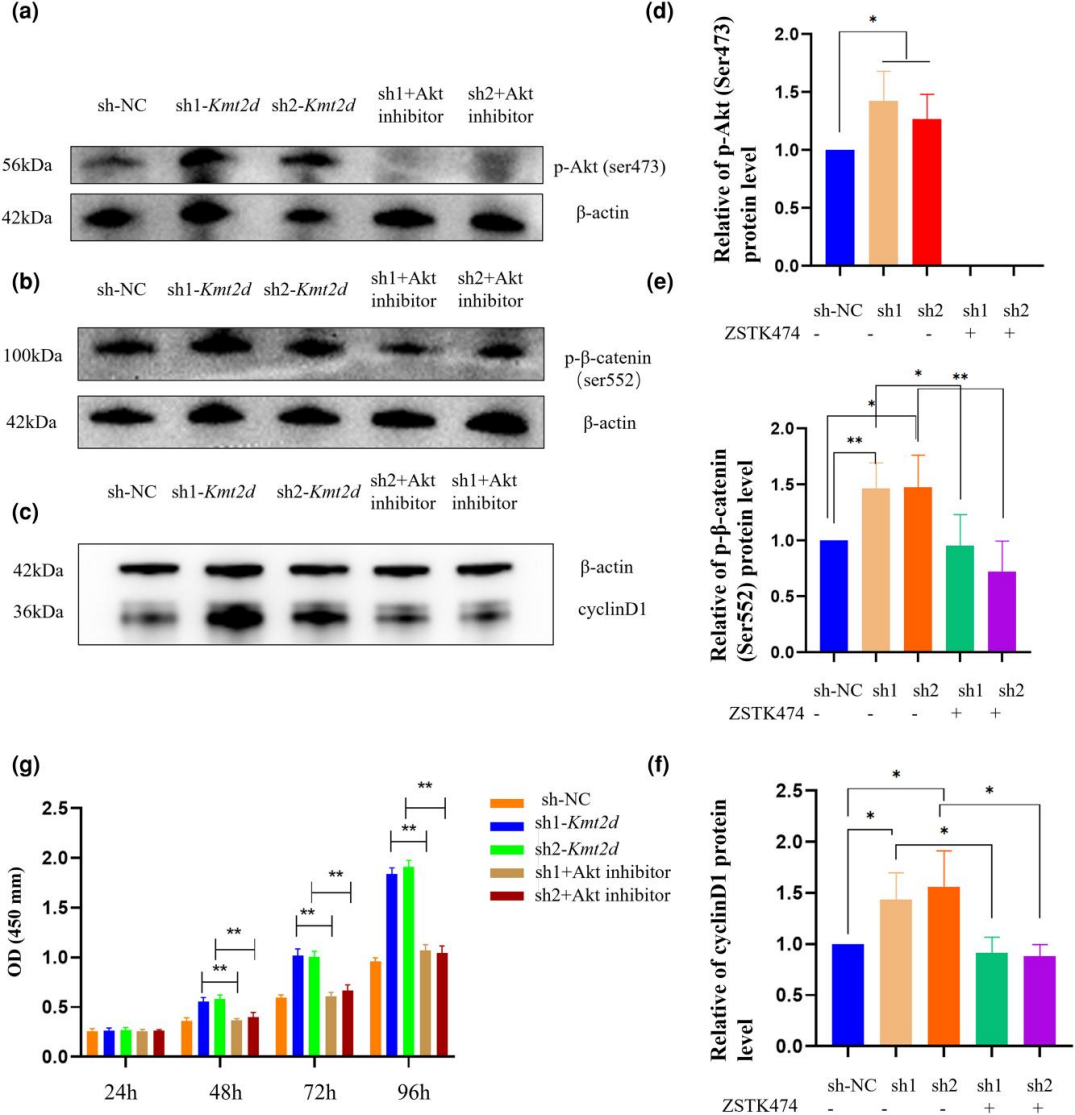
The effect of Akt inhibition on β-catenin signaling in the group of *Kmt2d* knockdown chondrocytes. a–f) Cells in the groups of sh1-*Kmt2d* and sh2-*Kmt2d* were pretreated with ZSTK474 (5 μmol). The cell lysates of each group were prepared and probed for p-Akt (Ser473), p-β-catenin (Ser552), and cyclin D1 by western blot. The band density was quantified and expressed as the relative gray value (compared with the control) by arbitrarily setting the control value as 1. Data were pooled from 3 independent experiments, and the results were represented as mean ± SD. g) The enhanced cell proliferation caused by a low expression of *Kmt2d* could be partly reversed by Akt inhibition. *P < 0.05, **P < 0.001.

To further investigate the involvement of the Akt/β-catenin signaling pathway, ATDC5 cells in the sh1-*Kmt2d* and sh2-*Kmt2d* groups were treated with 5 μmol/mL ZSTK474 (an Akt inhibitor, Beyotime) for a duration of 12 h. The results depicted in Fig. 4a–g demonstrate that the effects of reduced *Kmt2d* expression on Akt-induced transcriptional factor p-β-catenin (ser552), cyclin D1, and cell proliferation were reversed upon the administration of ZSTK474. These observations indicate that *KMT2D* may be positioned upstream, rather than downstream, of the Akt/β-catenin signaling pathway. In summary, the data presented in this study indicate that the knockdown of *Kmt2d* resulted in enhanced cell proliferation through the activation of the Akt/β-catenin signaling pathway.

### Inhibitor of Akt partially rescues the growth delay and chondrocyte malformation caused by *kmt2d* knockdown

**Fig. 5.**
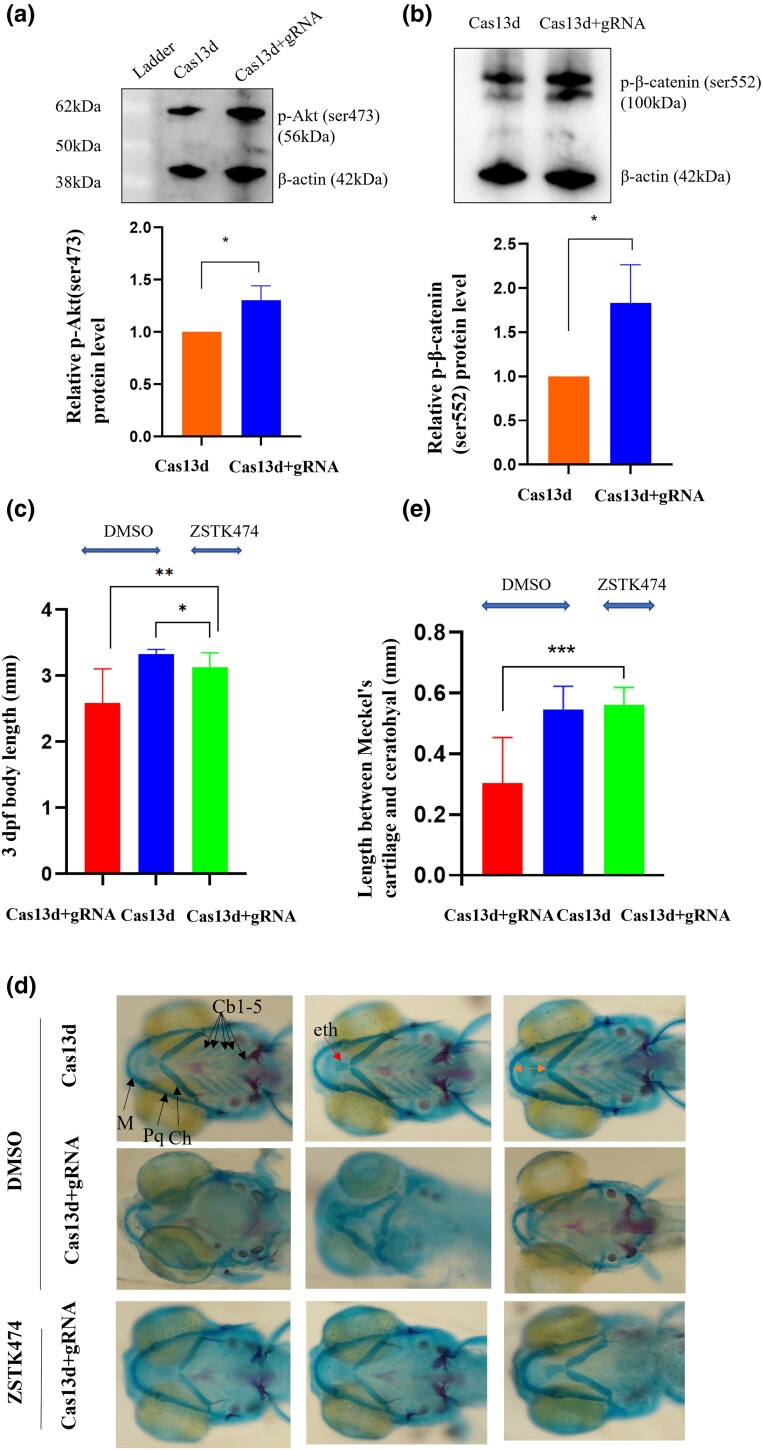
The growth delay and chondrocyte malformation induced by *kmt2d* deficiency can be partially alleviated by the inhibition of Akt. a and b) The levels of the p-Akt (ser473) and p-β-catenin (ser552) proteins. The band density was quantified and expressed as the relative gray value (compared with the control) by arbitrarily setting the control value as 1. Data were pooled from 3 independent experiments, and the results were represented as mean ± SD. c) The reduced body length in *kmt2d* knockdowns was partially alleviated by ZSTK474. *n* = 10 for each group. d and e) The asymmetrical morphology ceratohyal and Meckel’s cartilage in *kmt2d* knockdowns was partially corrected. *n* = 10 for each group. Scale bar: d) 100 μm. *P < 0.05, **P < 0.001, ***P < 0.0001. Cb, ceratobranchial; Ch, ceratohyal; M, Meckel’s cartilage; Pq, palatoquadrate.

We then asked whether p-Akt (ser473) and p-β-catenin (ser552) were affected in *kmt2d* deficiency embryos and the pharmacological inhibition of Akt/β-catenin signaling could rescue the growth delay and chondrocyte malformation caused by *kmt2d* knockdown. Western blot was used to detect p-Akt (ser473) and p-β-catenin (ser552), and the expression levels of both increased in the knockdown group compared with the control group (Fig. 5a and b). Zebrafish with the knockdown of *kmt2d* were subjected to treatment with a concentration of 5 μmol/mL of ZSTK474 from 24 hpf to 5 dpf, and 0.1% of the DMSO control was utilized. We observed that the diminished body length in *kmt2d* knockdowns was partially mitigated by the administration of ZSTK474, as depicted in Fig. 5c. Alcian blue staining of 5 dpf *kmt2d* knockdowns treated with ZSTK474 showed a rescue of the lower jaw in most embryos (Fig. 5e; Meckel’s cartilage and palatoquadrate; 9/10). Similarly, the average distance length between Meckel’s cartilage and ceratohyal also demonstrated a rescue effect, which was attributed to the rescue of the cartilage (Fig. 5d and Fig. 5e; *n* = 10). Similarly, the fin ceratobranchial elements of 5 dpf *kmt2d* knockdowns treated with ZSTK474 showed rescue in some embryos (Fig. 5d; Cb1-5; 6/10). In addition, the *kmt2d* knockdowns exposed to the compounds showed a recovery of craniofacial structures in a few embryos (Fig. 5d; 4/10). Thus, the inhibition of Akt in the *kmt2d* knockdowns could partially rescue the growth impairment and aberrant cartilage phenotypes observed in the zebrafish model of KS.

## Discussion

Short stature is a common feature in individuals with KS (Barry *et al*. 2022). Because rare diseases are extreme manifestations of genetic disorders, a study of these cases will help elucidate the understanding of the mechanism of human disease and promote potential new treatments. In this zebrafish and chondrocyte study, expression and functional studies were undertaken to explore the mechanism of low expression of the *Kmt2d* gene leading to short stature through an activated Akt/β-catenin signal pathway.

The CRISPR/Cas13d system can serve as an effective additional knockdown tool in zebrafish embryos. This study found that the CRISPR/Cas13d system can disrupt *kmt2d* function in the zebrafish embryonic model. Previous investigations have demonstrated that the administration of Morpholino (MO)s specifically targeting *kmt2d* mRNA resulted in the development of craniofacial abnormalities in zebrafish embryos (Bögershausen *et al*. 2015; Van Laarhoven *et al*. 2015). Similarly, embryos co-injected with Cas13d and 3 gRNAs targeting *kmt2d* mRNA displayed a significant reduction in *kmt2d* mRNA levels and H3K4me3 protein level, as well as related craniofacial defects. Interestingly, cranium bones, such as ethmoid plate, have defects akin to those reported in *kmt2d* MO-injected embryos (Van Laarhoven *et al*. 2015). Specifically, visceral cartilage anomalies in embryos co-injected with Cas13d plus *kmt2d* mRNA gRNAs were also observed in *kmt2d* MO-injected embryos (Bögershausen *et al*. 2015). Finally, similar to previous *kmt2d* MO-treated experiments, we also detected a shorter distance between Meckel’s cartilage and ceratohyal when *kmt2d* mRNA was targeted using Cas13d (Bögershausen *et al*. 2015). The results of this study provide further basis for studying the etiology of developmental defects in KS by using a CRISPR/Cas13d system to disrupt the *kmt2d* gene based on RNA editing.

During the process of endochondral ossification, bones are formed through the development of cartilage templates. Chondrocytes within these templates undergo proliferation and differentiation, eventually transforming into hypertrophic chondrocytes. Over time, these hypertrophic chondrocytes are gradually replaced by bone (Wuelling and Vortkamp 2010). Disruption of the G1/S checkpoint is a critical step in chondrocyte proliferation and skeletal growth (Stegen *et al*. 2022). KMT2D has been identified as a regulator of proliferation and cell cycle with varying outcomes depending on the specific cell type. Pang *et al*. (2021) conducted a study in which they observed that a knockdown of the *Kmt2d* gene resulted in a reduction in proliferation and induced a G1 phase arrest in dental epithelial cells. This effect was achieved through the activation of the Wnt/β-catenin signaling pathway. The upregulation of cyclin D1 and subsequent promotion of proliferation were observed in oral squamous cell carcinoma cells upon an overexpression of *Kmt2d* (Wang *et al*. 2022). Conversely, the conditional deletion of *Kmt2d* in mice during early B-cell development resulted in an enhanced proliferation of B cells (Zhang *et al*. 2015). Additionally, it was observed that cutaneous squamous cell carcinoma lacking KMT2D exhibited heightened cell proliferation and a faster G1/S transition in the cell cycle (Dauch *et al*. 2022). Our findings revealed a noteworthy enhanced proliferation in ATDC5 cells. The results of a flow cytometric analysis showed that the percentage of chondrocytes in the G1 phase was markedly decreased, whereas the percentage of chondrocytes in the S phase was significantly increased in *Kmt2d* gene knockdown cells compared with control cells. This leads to the inference that a knockdown of *Kmt2d* promotes proliferation via the promotion of G1/S progression in ATDC5 cells.

*Kmt2d* was shown to induce precocious chondrocyte differentiation in vitro, involving the loss of KMT2D-mediated H3K4me3 at the *Shox2* gene and release of *Sox9* inhibition (Fahrner *et al*. 2019). Our study focused on the impact of *Kmt2d* deficiency on chondrocyte proliferation, which is the initial and key process in endochondral ossification. However, the observation that the knockdown of *Kmt2d* resulted in an increase in both proliferation and differentiation of chondrocytes is paradoxical, as it is generally understood that cells undergo cell cycle arrest before entering the differentiation process. For example, pRb reduced proliferation and promoted chondrocyte differentiation by regulating cells exiting the cell cycle (Vale-Cruz *et al*. 2008). Additionally, deletion of *Cdk1* inhibits the proliferation and accelerates the differentiation of chondrocytes (Saito *et al*. 2016). Perhaps, KMT2D has dual roles in chondrogenesis, promoting cell proliferation while enhancing differentiation. This concept is buttressed by the action of other genes, which affect both cartilage proliferation and differentiation. For example, *Cdc5l* enhanced proliferation by inhibiting Wee1 expression along with eliciting differentiation in ATDC5 cells; both of these effects were mediated by modulating pre-mRNA splicing (Jokoji *et al*. 2021). Secondly, the overexpression of the *Flrt2* gene in ATDC5 cells resulted in an accelerated proliferation rate and enhanced chondrogenic differentiation (Xu *et al*. 2011). Finally, neuropeptide W can promote proliferation and differentiation in ATDC5 cells (Wang *et al*. 2019). As the physiological role of KMT2D in the endochondral ossification process in vivo remains unresolved, conducting studies on cartilage-specific *Kmt2d* knockout mice or zebrafish studies could provide invaluable insights. The mechanism by which KMT2D coordinates chondrocyte proliferation and differentiation during endochondral bone development remains elusive.

As described previously, both Akt and β-catenin were frequently dysregulated in mice with dwarfism and delayed bone development (Chen *et al*. 2001; Xia *et al*. 2019). In our study, we focused on the role of Akt/β-catenin in the effects of KMT2D on chondrocyte proliferation. β-Catenin plays a pivotal role in the proliferation of chondrocytes and the formation of the skeletal system (Xia *et al*. 2019). The western blot results revealed that the knockdown of the *Kmt2d* gene induced β-catenin activation and caused an upregulation of its targets including cyclin D1. Akt, a prosurvival factor, has garnered significant attention in the scientific community due to its central role in various physiological processes and pathological conditions. Previous studies have established the function of Akt in chondrocytes during endochondral ossification. The deletion of Akt1 resulted in a delay in calcification, whereas the activation of Akt in embryonic chondrocytes facilitated chondrocyte proliferation and hindered hypertrophic differentiation (Ford-Hutchinson *et al*. 2007). In addition, there is evidence that a study also indicated the regulation of β-catenin by Akt in chondrocyte cells (Wang *et al*. 2010). Phosphorylation of β-catenin by Akt facilitates its nuclear localization and enhances transcription of its target genes. By extension, further experiments are necessary to further elucidate the role of the Akt/β-catenin pathway as a mediator between KMT2D and chondrocyte development.

### Conclusion

Our results explored the role of KMT2D protein in chondrocyte functions and demonstrated, for the first time, that KMT2D modulates bone growth conceivably via regulation of the Akt/β-catenin pathway.

## Supplementary Material

jkad298_Supplementary_Data

## Data Availability

The data supporting the findings of this study are available within the article and supplementary material. The data that support the findings of this study are made available. Supplemental material available at G3 online.
