# Supplementary material for: Knockdown of Kmt2d leads to growth impairment by activating the Akt/β-catenin signaling pathway
Source: G3 (Bethesda). 2024 Jan 23;14(3):jkad298. doi: 10.1093/g3journal/jkad298 (PMC10917512; doi:10.1093/g3journal/jkad298)
Supplement: jkad298_Supplementary_Data [file jkad298_supplementary_data.docx]

**Supplemental data**

**Knockdown of *Kmt2d* led to growth impairment through the activating Akt/β-catenin signaling pathway**

Huakun Shangguan, Xiaozhen Huang, Jinduan Lin, Ruimin Chen

| \| 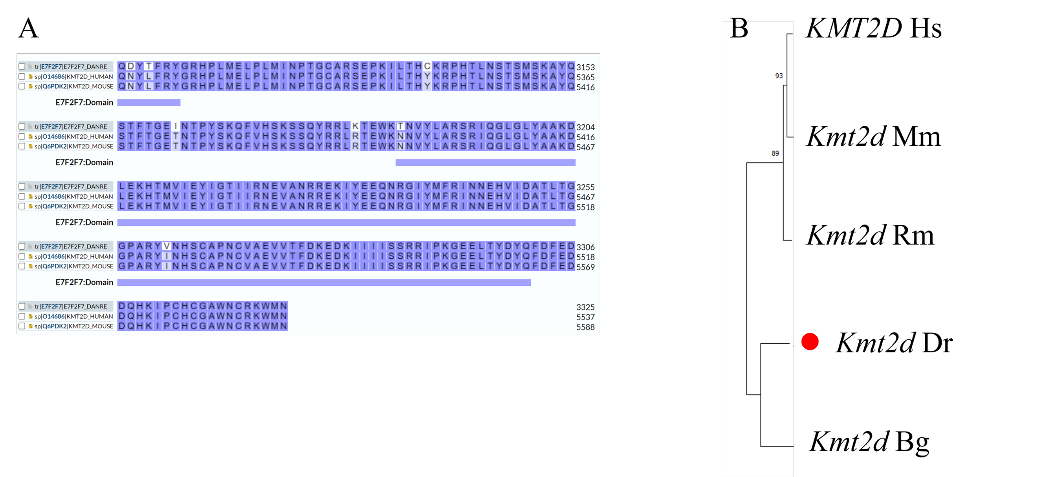 \| \| --- \| \| **Fig S1. Zebrafish *kmt2d* is highly conserved across multiple species.** (A) Multiple sequence alignments for coding sequence and amino acids of SET domain in KMT2D. (B) An evolutionary tree analysis of *kmt2d* (red dot presents zebrafish; Hs: human, Mm: mouse, Rm: Rhesus monkey; Dr: Danio rerio (zebrafish), Bg: Bufo gargarizans). \| |
| --- | --- | --- |

| 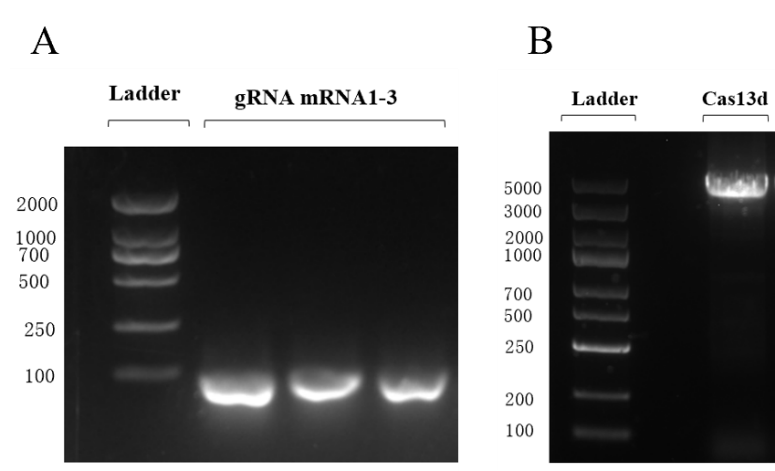 |
| --- |
| **Fig S2. The efficacy of *kmt2d* knockdown by CRISPR-Cas13d in zebrafish.** (A and B) RNA integrity of three gRNAs and Cas13d checked on Agarose Gel Electrophoresis. |
